# A Novel *de Novo* Mutation in the CD40 Ligand Gene in a Patient With a Mild X-Linked Hyper-IgM Phenotype Initially Diagnosed as CVID: New Aspects of Old Diseases

**DOI:** 10.3389/fped.2018.00130

**Published:** 2018-05-04

**Authors:** Tábata T. França, Luiz F. B. Leite, Tiago A. Maximo, Christiane G. Lambert, Nuria B. Zurro, Wilma C. N. Forte, Antonio Condino-Neto

**Affiliations:** ^1^Department of Immunology, Institute of Biomedical Sciences, University of São Paulo, São Paulo, Brazil; ^2^Immunodeficiency Sector, Department of Pediatrics, Irmandade da Santa Casa de Misericórdia de São Paulo, São Paulo, Brazil; ^3^Immunology Discipline, Santa Casa de São Paulo School of Medical Sciences, São Paulo, Brazil

**Keywords:** CD40 ligand, X-linked hyper-IgM syndrome, hypomorphic mutation, genetic defects, primary immunodeficiency

## Abstract

Mutations in the CD40 ligand (CD40L) gene (*CD40LG*) lead to X-linked hyper-IgM syndrome (X-HIGM), which is a primary immunodeficiency (PID) characterized by decreased serum levels of IgG and IgA and normal or elevated IgM levels. Although most X-HIGM patients become symptomatic during the first or second year of life, during which they exhibit recurrent infections, some patients exhibit mild phenotypes, which are usually associated with hypomorphic mutations that do not abrogate protein expression or function. Here, we describe a 28-year-old man who initially presented with recurrent infections since the age of 7 years, when he exhibited meningitis caused by *Cryptococcus neoformans*. The patient had no family history of immunodeficiency, and based on clinical and laboratory presentation, he was initially diagnosed with common variable immunodeficiency (CVID). In subsequent years, he displayed several sporadic episodes of infection, including pneumonia, pharyngotonsillitis, acute otitis media, rhinosinusitis, fungal dermatosis, and intestinal helminthiasis. The evaluation of CD40L expression on the surface of activated CD3^+^CD4^+^ T cells from the patient showed decreased expression of CD40L. Genetic analysis revealed a novel *de novo* mutation consisting of a 6-nucleotide insertion in exon 1 of *CD40LG*, which confirmed the diagnosis of X-HIGM. In this report, we describe a novel mutation in the CD40L gene and highlight the complexities of PID diagnosis in light of atypical phenotypes and hypomorphic mutations as well as the importance of the differential diagnosis of PIDs.

## Introduction

Hyper-IgM (HIGM) syndromes are a heterogeneous group of primary immunodeficiency disorders (PID) caused by defects in immunoglobulin (Ig) class switch recombination (CSR), with or without somatic hypermutation (SHM) defects (1, 2). Through these mechanisms, B cells induce a switch in immunoglobulin from IgM to IgG, IgA, or IgE and enhance the affinity of immunoglobulins for antigens (3). Many molecules are involved in these processes, and disruption of some of these processes can cause elevated serum levels of IgM, which are typically associated with HIGM syndromes but have different clinical diagnoses depending on the mutated gene (4, 5).

In immunoglobulin class switching, an initial signal results from the interaction between CD40L/CD40, ICOS/ICOSL, and BAFF-APRIL/TACI, which are expressed by T and B cells. A second signal is given by the following cytokines synthesized by Th1, Th2, and Th3 cells: IFN-γ, resulting in class switching to IgG; IL-4 and 13, resulting in switching to IgE; and IL-5, IL-10, and TGF-β, resulting in switching to IgA (6). Thus, the interaction between CD40L and CD40 provides a fundamental signal for B cell proliferation and differentiation that leads to CSR and SHM (1, 3). In addition to controlling immunoglobulin class switching in B cells, CD40L-CD40 interactions have been implicated in various biological processes, such as the maturation and activation of different cell types and granulopoiesis in the bone marrow (7).

X-linked hyper-IgM syndrome (X-HIGM; HIGM type 1; OMIM # 308230) is a rare PID characterized by normal or elevated serum levels of IgM in association with decreased levels of IgG, IgA, and IgE, (8, 9). X-HIGM results from mutations in the CD40 ligand gene (*CD40LG*), which is located on chromosome Xq26.3 (10–14). *CD40LG* encodes the membrane glycoprotein CD40 ligand (CD40L, CD154 or gp39), which is mainly expressed on the surface of activated T cells and platelets (15).

X-HIGM patients are susceptible to pneumonia, recurrent upper and lower respiratory tract infections, chronic neutropenia, gastrointestinal manifestations and autoimmune diseases (8, 9, 16, 17). Patients with classical X-HIGM become symptomatic during the first or second year of life, during which they exhibit sinopulmonary and opportunistic infections, usually caused by *Pneumocystis jirovecci, Cryptosporidium* spp. and *Cryptococcus* spp. (4, 8, 17–22), although other infections may be present depending on pathogen exposure and geographic location (4). In contrast to classical X-HIGM, some patients display milder phenotypes, characterized by late onset of symptoms, few severe complications and good responses to intravenous immunoglobulin (IVIg) therapy (5, 16). Bone marrow transplantation may be necessary in cases of CD40L deficiency with uncontrollable opportunistic infections (23).

Mild phenotypes are usually associated with hypomorphic mutations that do not abrogate protein expression and function, which makes the diagnosis more difficult to confirm (16, 24–26). Here, we describe a 28-year-old man who exhibited recurrent infections beginning at the age of 7 years. He had no family history of immunodeficiency, and based on his clinical and laboratory presentations, he was initially diagnosed with common variable immunodeficiency (CVID). Evaluation of CD40L expression on the surface of activated CD3^+^CD4^+^ T cells revealed decreased expression of CD40L, and a genetic analysis revealed a novel mutation in the CD40L gene (*CD40LG*), which confirmed the diagnosis of X-HIGM.

## Case report

A 13-year-old boy was referred to the Immunology Clinic due to the following severe recurrent infections: treatment-resistant pneumonia at the age of 12 years and meningitis caused by *Cryptococcus neoformans* at the age of 7 years (Table 1). He was well and exhibited no signs or symptoms of infection, and he showed no changes upon physical examination. The patient's previous history included detachment of the umbilical stump in <20 days, no adverse reactions to vaccines, and no serious infections or hospitalizations up to his 7 years of age. The parents were non-consanguineous and healthy. The patient was a third-pregnancy child with no family history of immunodeficiency nor recurrent infections or serious diseases.

**Table 1:** Laboratory examination of cerebrospinal fluid.

**Analysis performed**	**Test result**	**Normal value**
India ink test	Positive	Negative
Latex agglutination test	Positive	Negative
Macrophages with internalized cryptococcus	2%	0%
Cryptococcus	15/mm^3^	0%
Protein	22 mg/dL	<40 mg/dL
Glucose	47 mg/dL	48–74 mg/dL
Cells	09/mm^3^	<4/mm3
Monocytes	29%	30–50%
Lymphocytes	69%	50–70%
Neutrophils	0%	0%

At the Immunology Clinic, laboratory tests revealed the following: negative serology for C-reactive protein (CRP), human immunodeficiency virus (HIV), Epstein-Barr virus (EBV), Cytomegalovirus (CMV) and toxoplasmosis. His serum immunoglobulin levels were normal for IgM, while decreases in IgA, IgE and IgG were observed (IgM, 193 mg/dL, IgA, <7 mg/dL, IgE, <17 mg/dL, and IgG, 230 mg/dL). The other immunological examinations showed normal results for the total T lymphocyte count, CD19, CD4, CD8, CH50, C3, and C4 levels, chemotaxis, NBT, and neutrophil and mononuclear phagocytic ingestion. Based on the clinical and laboratory findings, the initial hypothesis was CVID. Considering that the patient was well and showed no clinical manifestations or infections at the time and that his IgG was >200 mg/dL, the clinical team opted for a clinical laboratory follow-up, with monthly outpatient observation.

During the course of the next 2 years of follow-up, the patient remained asymptomatic. However, in subsequent years, he began to present sporadic episodes of infections, including pharyngotonsillitis (2 episodes), acute otitis media (2), rhinosinusitis (3), pneumonia (3 episodes, 1 every 2 years), fungal dermatosis (5), and intestinal helminthiasis (2). All of these infections were easily treated, and good resolutions were obtained using oral antibiotics and topical antifungal or vermifuge treatments. His immunoglobulin profile continued to show reduced levels of IgG and IgA (197 and <25 mg/dL, respectively). However, his IgM levels increased (374 mg/dL), which elicited the hypothesis of HIGH syndrome.

At age 22, the patient presented with treatment-resistant pneumonia that required broad-spectrum treatment and hospitalization for 5 days. On this occasion, a change in his laboratory profile was observed, as follows: serious reduction of IgG (<93 mg/dL) and IgA (<0.7 mg/dL) levels and very high levels of IgM (625 mg/dL). Due to these alterations, he was prescribed human IVIg replacement therapy at 500 mg/kg/month. X-HIGM syndrome due to a CD40L defect was suspected, and this hypothesis was subsequently confirmed. The patient's immunoglobulin profile over the years is summarized in Table 2.

**Table 2:** The patient's serum immunoglobulin levels (mg/dL) over the years.

**Immunoglobulin isotype**	**2002 (13 year)**	**2006 (17 year)**	**2011 (22 year)**	**Normal value**
IgM	193	374	625	30–212
IgG	230	197	<92.7	600–2.120
IgA	<7	<25	<0.7	80–476
IgE	<17	<17	<19.2	

During the course of the last 6 years, he has continued to receive IVIg, with serum IgG > 500 mg/dL being observed before each administration. He presented with 4 episodes of rhinosinusitis and one episode of onychomycosis, all of which were resolved after the administration of oral medications, and no recurrence of pneumonia or opportunistic infections was observed.

**Figure 1:**
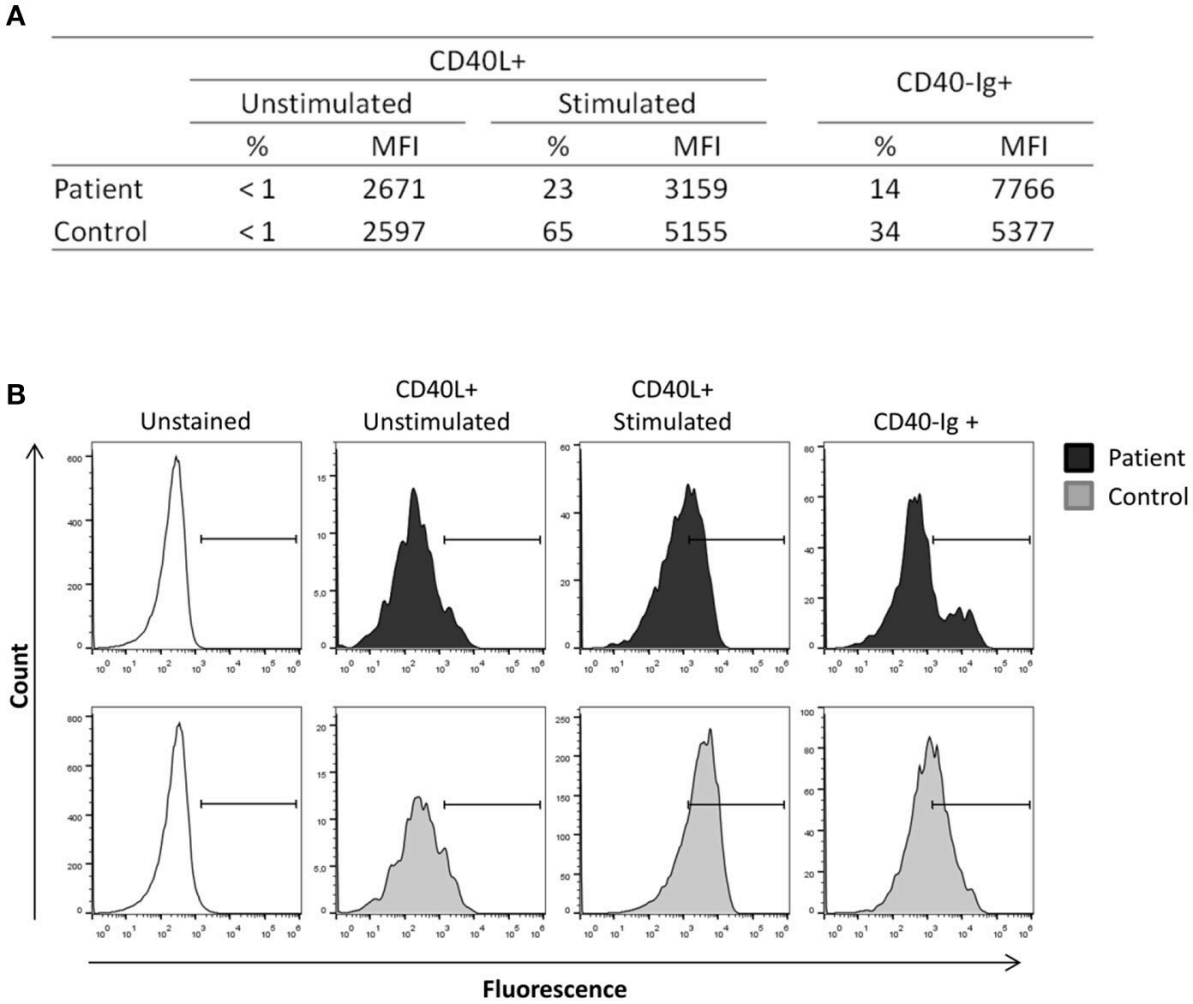
CD40L expression in activated lymphocytes. Peripheral blood mononuclear cells from the patient and a healthy control were stimulated with ionomycin (1 μg/mL) and PMA (20 ng/mL) for 3 h, and non-adherent cells were then collected, stained and analyzed by flow cytometry. CD40L expression and CD40Ig binding were analyzed in CD3+CD4+ gated cells. **(A)** The patient exhibited reduced expression of CD40L after stimulation compared with that seen in the healthy control and displayed reduced CD40-Ig binding. **(B)** Representative histograms showing the reduction of CD40L expression and CD40Ig binding in the patient. The experiments were performed in duplicate and repeated twice with two distinct healthy controls.

To confirm the diagnosis of X-HIGM, the expression of CD40L was evaluated. CD40L expression on the surface of resting and PMA/ionomycin-activated CD3^+^CD4^+^ peripheral blood lymphocytes from the patient was analyzed by flow cytometry. Additionally, the binding of CD40L to CD40-muIg, which is a fusion protein consisting of the extracellular domain of human CD40, was assessed to evaluate the functional properties of CD40L. Although the patient exhibited normal activation of CD3^+^CD4^+^ lymphocytes, as indicated by expression of the activation marker CD69 (data not shown), he showed a reduced frequency of CD40L expression after stimulation compared with that seen in the healthy control (23 and 65%, respectively; Figure 1A). In accordance with the reduced expression of CD40L, the binding of CD40-Ig to CD40L was reduced in the patient compared with that seen in the healthy control (14 and 34%, respectively; Figure 1A). Representative histograms are shown in Figure 1B.

**Figure 2:**
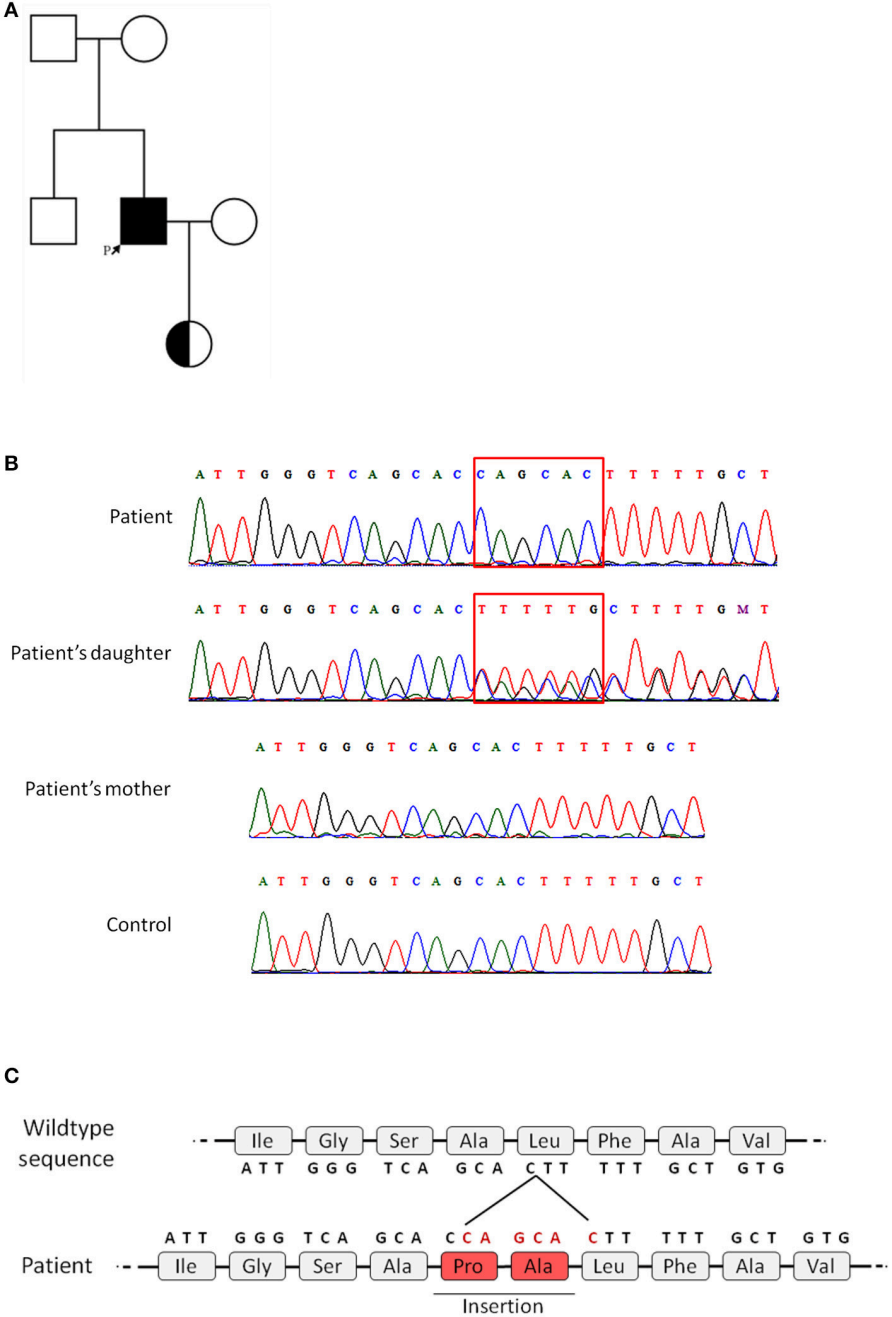
Family pedigree and genetic analysis of the CD40L gene. Genetic analysis was performed on the patient and the patient's daughter and mother by Sanger sequencing. All five exons of *CD40LG* were analyzed. **(A)** Family pedigree (the patient is indicated by the arrow). **(B)** Chromatogram showing the patient's 6-nucleotide insertion in exon 1 and the heterozygous genotype of the patient's daughter, in contrast to the patient's mother and a healthy control. **(C)** Representation of the predicted insertion of 2-amino acids in the sequence of the mutated protein.

Because the patient exhibited reduced, but not absent CD40L expression, identification of the genetic mutation in the *CD40LG* was necessary to achieve a definitive diagnosis. Genetic analysis performed through Sanger sequencing of the *CD40LG* revealed a 6-nucleotide insertion in exon 1 of *CD40LG* (c.121_122insCAGCAC), thus confirming the diagnosis of X-HIGM (Figure 2B). Notably, the inserted sequence, CAGCAC, was a duplication of the sequence immediately before the insertion and *in silico* analysis performed with Mutation Taster predicted the addition of two amino acids (proline and alanine) to the protein sequence (Figure 2C). The patient's mutation was not found in any of the databases that were searched (dbSNP, ExAC, Genome Mutation, and 1,000 Genomes Project), which confirmed that it was a novel mutation. Because the mutation found in the patient was located on the X chromosome, genetic analysis was performed on the patient's daughter, and she was found to be heterozygous for the mutation, as expected (Figure 2B). To investigate the origin of the mutation, a genetic analysis was also performed on the patient's mother, which revealed that she did not carry the mutation found in the patient; thus, this mutation is a novel *de novo* mutation (Figure 2B). The family pedigree is presented in Figure 2A.

## Discussion

In addition to the heterogeneity of HIGM syndromes, hypomorphic mutations that impair, but do not abrogate protein function and expression may lead to atypical presentations of each disease (27). Hypomorphic or milder mutations that allow binding of CD40L with CD40 have been reported to be associated with a less severe clinical course (5, 16, 24–26, 28, 29). Here, we report the case of a 28-year-old Brazilian man who exhibits a mild X-HIGM phenotype including late-onset symptoms, which began with meningitis caused by an opportunistic pathogen at the age of 7 years and bacterial pneumonia at 12 years. The initial diagnosis was CVID, in view of the findings of decreased IgG, IgA, and IgE levels, associated with normal IgM levels and B lymphocyte counts. Subsequently, the patient presented with increased levels of IgM, suggesting a diagnosis of X-HIGM, which was confirmed thereafter. Consistent with the atypical picture of mild phenotypes, the patient exhibited a good response to IVIg therapy, and no severe or persistent infection was reported after the treatment began.

Several humoral immunodeficiencies are associated with hypogammaglobulinaemia and recurrent sinopulmonary infections, among which the most clinically relevant deficiencies usually present in infancy or childhood (30). Indeed, in 90% of cases, the initial clinical presentation of X-HIGM occurs during early childhood up to 4 years of age (2, 17). However, in the last few years, due to advancements in genetic analyses, accurate diagnoses of immune function disorders have been achieved, and milder phenotypes of previously known severe genetic defects have been revealed (4), which has increased the number of described cases with late onset, as in the case presented here. This pattern highlights the fact that X-HIGM can first manifest late in the life with a mild phenotype and should be considered in the differential diagnosis of opportunistic infections in non-HIV-infected patients (26).

The defects in immunoglobulin production presented by CVID patients are not always discernible from other humoral immunodeficiencies, such as HIGMs. Moreover, some patients with CVID may present T cell activation defects resulting in an HIGM-like phenotype, including reduced expression of CD40L (31, 32). The clinical and immunological overlap between CVID and HIGM makes the diagnosis more difficult to confirm, which highlights the importance of differential diagnosis in clinical practice. One aspect that should be considered in the differential diagnosis of X-HIGM, as in other PIDs, is susceptibility to specific classes of pathogens. In the present case, the first manifestation was meningitis caused by an opportunistic agent (*Cryptococcus neoformans*). Infections caused by this intracellular pathogen have been reported in X-HIGM patients by others (8, 18, 33) and are responsible for approximately 7% of central nervous system infections (34). In contrast, patients with CVID appear to be particularly susceptible to encapsulated (e.g., *Haemophilus influenza* and *Streptococcus pneumoniae*) and oratypical (e.g., *Mycoplasma* sp. and *Ureaplasma* sp.) bacteria (30, 35).

In accordance with the clinical presentation of the patient and laboratory findings, he exhibited reduced expression of CD40L in activated CD3^+^CD4^+^ T cells compared with that seen in healthy controls, and a mutation in exon 1 of *CD40LG* was found, which was later characterized as a novel *de novo* mutation, as his mother did not harbor the mutation. The CD40L molecule consists of four distinct structural domains: an N-terminal intracellular trail, a short transmembrane domain, a unique extracellular domain and an extracellular C-terminal TNF-homologous domain (36). Human *CD40LG* consists of five exons: exon 1 encodes the intracellular, transmembrane and a small portion of the extracellular region, whereas exons 2–5 encode the rest of the extracellular domain (GenBank no. D31793-D31797). The mutation found in our patient was a 6-nucleotide insertion in exon 1 of *CD40LG*, which encodes the transmembrane region. The majority of mutations in *CD40LG* occur in the region encoding the extracellular domain of the protein (approximately 90%, available at the CD40L database: http://structure.bmc.lu.se/idbase/CD40Lbase/index.php), and only a few mutations in the transmembrane portion of CD40L have been reported (5, 13, 37–42). All of the reported mutations in the region encoding the transmembrane region are associated with a lack of CD40L expression on the cell membrane surface, due to splice site mutations (5) or single nucleotide variants leading either to a premature stop codon (5, 37, 41) or a change in the amino acid sequence resulting in a new positive charge in the transmembrane domain (5, 13, 38–40, 42).

Indeed, the introduction of a positively charged amino acid into the membrane-spanning portion of the protein may cause problems in the stability of the helix or its insertion into the membrane (36, 43). Additionally, positively charged substitutions in the transmembrane helices may act as signals that guide the protein to be degraded in the endoplasmic reticulum (43). In our patient, the insertion of six nucleotides into the DNA sequence was predicted to cause the addition of two amino acids (proline and alanine) in the protein sequence. As the amino acids that were added to the protein were both non-polar with neutral charges, the reduced expression of CD40L in the cell membrane may not have been related to the introduction of positively charge residues in the transmembrane region. Impairment of the efficiency of the maturation of translated proteins on the cell surface, protein instability caused by the mutated gene, and even protein retention in the endoplasmic reticulum are possibilities that have been raised in previous work to justify decreased expression of CD40L on the cell surface caused by mutations affecting the transmembrane region of the molecule (38), which could explain the reduced CD40L expression observed in our patient.

An interesting case of a mutation in exon 1 that affect the intracellular domain of CD40L (p.R11X) and result in the generation of a mutant CD40L predicted to lack the cytoplasmic domain that is still capable of CD40-binding are associated with impaired expression of CD40L and a milder phenotype (24, 26, 44, 45). Because the extracellular domain of CD40L (which binds to the CD40 receptor) is not affected in our patient, it is likely that the expressed CD40L is able to interact with CD40, resulting in some residual activity, contributing to the immune response; however, the reduced surface expression may have been insufficient for proper, effective activation, leading to increased susceptibility to infections. The crystallographic structure of the transmembrane domain of CD40L is not available in the literature, which makes it not possible to further study the effects of the mutation on the protein structure. Further detailed analysis of the mutation and its effect on the expression and function of CD40L will be needed to confirm our hypothesis and understand the mechanisms underlying the mutation as well as its phenotypic consequences.

The similarities of clinical manifestations, laboratory presentation, and immunological defects observed in CVID and other PIDs, such as HIGM syndromes, may lead to a misdiagnosis (46, 47). In fact, several groups described patients affected by PIDs due to distinct mutations that were initially misdiagnosed with CVID before receiving a correct diagnosis (48–51). Although the clinical outcomes are similar in CVID and other PIDs, the genetic basis is different and an accurate diagnosis improves therapeutic approaches, which significantly reduces the frequency and severity of infections in affected individuals from childhood, and allows for proper genetic screening and counseling of the patient's family. In this regard, an incorrect or late diagnosis directly affects the patient's long-term outcomes and quality of life. Moreover, early diagnosis shortens diagnostic delay that is distressing to the family, damaging to the patient and waste of health-care resources (52).

In conclusion, our data suggest that the reduced expression of CD40L on the CD3^+^CD4^+^ T cell surface in the patient described herein is a consequence of the identified mutation in *CD40LG*, proving the basis for the milder phenotype presented by the patient. The strong correlation between the patient's genotype, molecular data, and phenotype supports this hypothesis. Additionally, the present work highlights the complexities of PID diagnosis and the importance of differential diagnosis in clinical practice. The accurate diagnosis of affected patients provides useful information about disease prognosis, which enables specific laboratory monitoring and supporting therapeutic decisions, to avoid future complications. Furthermore, the genetic investigation and identification of female carriers allows proper genetic counseling and early genetic testing of children, as demonstrated in the reported case.

## Ethics statement

This study was carried out in accordance with the recommendations of the ethical committee of the University of São Paulo. The protocol was approved by the ethical committee of the University of São Paulo. All subjects gave written informed consent in accordance with the Declaration of Helsinki.

## Author contributions

TF, LL, and TM wrote the manuscript. LL and TM performed clinical and laboratory data collection and analysis. TF, CL, and AC-N provided data from functional testing and genetic analysis. LL, TM, and WF follow the patient. NZ contributed to the genetic analysis. AC-N, WF, and LL provided a critical revision of the article and gave final approval of the version to be published.

### Conflict of interest statement

The authors declare that the research was conducted in the absence of any commercial or financial relationships that could be construed as a potential conflict of interest.
